# Associations between pulse pressure amplification and inflammation in young adults according to body composition: The African-PREDICT study

**DOI:** 10.1038/s41371-026-01126-9

**Published:** 2026-02-27

**Authors:** Yolandi Breet, Christian Delles, Paul Welsh, Catharina MC Mels

**Affiliations:** 1 Hypertension in Africa Research Team (HART), North-West University, Potchefstroom, South Africa; 2 MRC Research Unit for Hypertension and Cardiovascular Disease, North-West University, Potchefstroom, South Africa; 3 School of Cardiovascular and Metabolic Health, University of Glasgow, Glasgow, UK

**Keywords:** Risk factors, Hypertension

## Abstract

Pulse pressure amplification (PPA) is a measure of arterial function and wave reflection dynamics. The potential contribution of low-grade systemic inflammation to PPA—particularly in the context of overweight and obesity (OW/OB)—remains unclear, especially in young adults. We assessed differences in PPA and inflammatory markers (leptin, interleukin-6, interleukin-8, tumour necrosis factor-α, adiponectin, interleukin-10, and C-reactive protein) in young adults stratified by body composition. We further determined the relationship of PPA and inflammatory markers within these groups. This cross-sectional study included 1202 adults aged between 20-30 years. Participants were stratified into groups with underweight (*N* = 82) healthy weight (*N* = 598), overweight (*N* = 326) and obesity (*N* = 196). PPA was defined as the ratio of brachial pulse pressure to central pulse pressure. Inflammatory biomarkers were measured in serum. PPA was lower in the OW and OB groups (*p* < 0.001), while the inflammatory profile (tumour necrosis factor-α, adiponectin, leptin and C-reactive protein) was also more adverse in the OW and OB groups (all *p* < 0.001). In multivariate adjusted regression analysis, PPA was adversely associated with tumour necrosis factor-α, adiponectin, leptin and C-reactive protein in the OW or OB groups only (all *p* < 0.025). In young adults, a higher BMI is associated with a lower PPA and higher levels of inflammatory markers. Adverse associations between PPA as a measure of arterial function and several inflammatory markers are also seen in the setting of increased adiposity, highlighting inflammation as a potential mechanistic link between adiposity and changes in arterial function.

## Introduction

Pulse pressure (PP) is regarded as a key indicator of arterial health and its role in cardiovascular risk is well established [1, 2]. Pulse pressure varies significantly throughout the arterial tree [3] due to an increase in arterial pressure as the pulse wave travels forward in the arterial system to less compliant regions [4]. Therefore, in healthy individuals, PP is amplified between the central and peripheral arteries [5] as a result of the progressive increase of arterial stiffness along the vascular tree [6]. The amplification effect between peripheral and central PP, is known as pulse pressure amplification (PPA) and is associated with increased cardiovascular risk [7].

Although PPA is influenced by a number of physiological factors, such as age, sex, height and heart rate [2, 8], as well as certain modifiable factors including smoking, hypercholesterolemia and diabetes [8–10], less is known about the role of inflammation and body composition in this regard. While studies have shown obesity to be related to adverse arterial changes [11–14], the exact mechanism is currently not fully elucidated. One such mechanistic link, could be the role of inflammation in vascular structure and function.

Obesity is often associated with low-grade chronic inflammation [15] marked by increased levels of systemic markers of inflammation such as C-reactive protein (CRP) [16]. Adipose tissue is also a significant source of inflammatory cytokines and other factors [17, 18]. Adiposity-related pro-inflammatory markers and lower levels of anti-inflammatory markers have been linked to adverse changes in vascular structure and function such as increased arterial stiffness, reflected by an increase in pulse wave velocity (PWV) [19–21], possibly explaining the link between obesity and detrimental arterial alterations. However, less is known about the role of inflammation and body composition in PPA, especially in young adults. We therefore aimed to assess differences in PPA as well as an array of inflammatory markers (leptin, interleukin-6, interleukin-8, tumour necrosis factor-α, adiponectin, interleukin-10, and C-reactive protein) in young adults stratified by body composition according to body mass index (BMI). We further determined the relationship of PPA and inflammatory markers within these groups.

## Methods

### Study population

This study forms part of the African Prospective study on the Early Detection and Identification of Cardiovascular Disease and Hypertension (African-PREDICT). The African-PREDICT study was designed to investigate the early pathophysiology accompanying CVD development and to identify novel early markers or predictors by following young, apparently healthy adults over a period of 10–20 years [22]. This sub study included the cross-sectional data from 1202 participants from the baseline cohort, with complete data for the variables of interest. Participants were recruited from Potchefstroom, South Africa, as well as the surrounding areas by field workers, via their workplace, or through advertisement by means of local newspapers or radio stations as well as word of mouth. Young, apparently healthy Black and White men and women between the ages of 20-30 years were initially screened and those who met the eligibility criteria were included in the study. The specific exclusion criteria were a mean office BP ≥ 140 mm Hg and/or 90 mm Hg, human immunodeficiency virus infection, previous diagnosis with any chronic disease, pregnancy, or breastfeeding.

This study complies with the requirements of the international ethics guidelines, the Declaration of Helsinki as well as the Department of Health (DoH) guidelines on research in human participants (2024). The study protocol was approved by the Health Research Ethics Committee of the North-West University (NWU-00001-12-A1). All procedures were explained to the participants prior to any measurements being taken and all participants gave written informed consent prior to enrolment. The African-PREDICT study is registered in a clinical trials registry (NCT03292094).

### Questionnaires

Participants completed a general health questionnaire with the help of a trained researcher where self-reported sex, ethnicity, age, alcohol consumption and tobacco use were recorded. The socioeconomic status (SES) score of a participant was derived from three categories included in the general health questionnaire, namely skill level (derived from occupation), education, and household income. Each category was scored, and the total score used to classify participants into low, middle, or high SES classes. This classification was adapted from Patro et al [23].

### Anthropometric measurements

Height (SECA 213 Portable Stadiometer, SECA, Hamburg, Germany) and weight (SECA 813 Electronic Scales, SECA, Hamburg, Germany), were measured using standardized methods and calibrated instruments [24], after which body mass index (BMI) was calculated. BMI was categorized according to the World Health Organization guidelines as follows: Underweight as below 18.5, healthy weight as 18.5-24.9, overweight as 25.0-29.9, and obesity as 30.0 or above. Waist circumference was measured three times using a non-flexible tape measure (Holtain, Crymych, UK) and recorded to the nearest 0.1 cm. The median of the three measurements was used in subsequent analyses. Waist-to-height ratio (WHtR) was subsequently calculated.

### Cardiovascular measurements

A validated blood pressure apparatus (Dinamap Procare 100 Vital Signs Monitor, GE Medical Systems, Milwaukee, USA) with appropriately sized GE Critikon latex-free Dura-Cuffs were used to measure office brachial blood pressure and heart rate. After a five-minute resting period, duplicate measurements were recorded on the left and right arm (with 5-minute intervals), with the arm rested at heart level and feet placed flat on the floor. The average of the second left and right arm measurement was calculated.

The SphygmoCor XCEL device (SphygmoCor XCEL, AtCor Medical, Sydney, Australia) was used, with the participant in supine position, to produce an arterial waveform that provided an estimated central systolic BP, central pulse pressure (cPP) and augmentation index (AIx) reading, obtained via the built-in generalized transfer function. This device was included in a large validation study of non-invasive cBP measurement techniques and was validated as an automated oscillometric monitor to assess cBP with acceptable accuracy [25].

The pulse wave analysis was also used to measure the supine brachial systolic- and diastolic blood pressure, from which mean arterial pressure (MAP) and brachial pulse pressure (bPP) were calculated. Pulse pressure amplification (PPA) was defined as the ratio of the amplitude of the PP between a distal and proximal location (bPP/cPP).

Pulse wave velocity (PWV) was captured at the right carotid and femoral arterial pulse points. The femoral artery wave form was captured via an appropriately sized cuff placed around the thigh, and the carotid arterial waveform was captured simultaneously via applanation tonometry. The distances between the pulsated sites were measured and 80% of these distances were used as the pulse wave travelled distance [26].

### Biological sampling and biochemical analyses

Participants were requested to fast from 22:00 the evening before the day of participation. Blood Samples were collected and immediately taken to the onsite laboratory where they were processed according to standardized protocol and aliquoted into cryovials for short- and long-term storage at –80 °C in bio-freezers.

Leptin, adiponectin, IL-6, and TNF-α levels were measured from serum with an enzyme-linked immunosorbent assay (ELISA) kit (R&D systems, Minneapolis, MN, USA), and analysed on a Synergy H4 hybrid microplate reader (BioTek, Winooski, VT, USA). A MILLIPEX Map Human High Sensitivity T Cell Magnetic Bead Panel (EMD Millipore, Merck, Missouri, USA) was used to measure IL-10 and IL-8. This panel was analysed using LuminexMAP technology on the Luminex 200^TM^ analyser which performed immunoassays on the surface of MagPlex-C microspheres, fluorescent-coded magnetic beads.

Low density lipoprotein cholesterol (LDL-C), high density lipoprotein cholesterol (HDL-C) and total cholesterol were also measured from serum whereas glycated haemoglobin (HbA1c) was determined from EDTA whole blood as well as sodium fluoride glucose, all of which were analysed using the Cobas Integra® 400 plus (Roche, Basel, Switzerland).

### Statistical analyses

In a-priori power analysis, using the G*power v3.1.9.3 software, a sample size was computed as a function of the required power level. The preselected power was 80% with the prescribed significance level estimated at α = 0.05. The population effect size was also detected at the probability of 1- β (in this case 0.6) for the main outcome measure of the original study. The priori analysis calculated that an N value or population of 72 per group would be sufficient for the hypothesis of this study.

Statistical analyses were performed with IBM® SPSS® Statistics version 29 software (IBM Corporation; Armonk, New York, USA). All variables were tested for normality by visual inspection (Q-Q plots) and evaluation of the Skewness and Kurtosis. We used analysis of variance to compare the characteristics of the study cohort stratified by BMI categories. Multiple linear regression analyses were performed between PPA, as well as PWV as main dependent variables and inflammatory markers, in the different groups. Covariates included age, sex, ethnicity, SES score, LDL, glucose, HR, smoking and alcohol use and was based on exploratory Pearson correlations. The models where PWV was the dependent variable, were additionally adjusted for MAP.

In supplementary analysis, the population was stratified into two groups based WHtR, with a normal WHtR regarded as < 0.5 and an increased WHtR as ≥ 0.5. Independent T-tests were performed to compare PPA, PWV and markers of inflammation between these two groups. Multiple linear regression analyses were also performed between PPA, PWV and inflammatory markers in the WHtR groups, with adjustment for age, sex, ethnicity, SES score, LDL, glucose, HR, smoking and alcohol use. The models where PWV was the dependent variable, were additionally adjusted for MAP.

## Results

Table 1 shows the characteristics of the study population stratified by BMI. The overweight (*p* < 0.001) and obesity (*p* < 0.001) groups were slightly older and presented with a higher socioeconomic status score (all *p* < 0.001). As expected, the body weight was higher in the overweight and obesity groups (*p* < 0.001), with WC also being higher in these groups (*p* < 0.001). With regards to the cardiovascular measurements, blood measures (SBP, DBP, MAP) were higher in the overweight and obese groups (all *p* < 0.001), with PPA being lower (*p* < 0.001). No differences in PWV between the groups were evident (*p* = 0.073). Markers of inflammation tended to be more adverse in the overweight and obesity groups with TNF-alpha, leptin and CRP being higher (all *p* < 0.001) and adiponectin lower (*p* < 0.001) in the overweight and obesity groups. No differences were encountered between the groups for interleukin-6, interleukin-8, or interleukin-10 (all *p* > 0.060).

**Table 1:** Basic characteristics of the study population stratified by body composition.

	Underweight (BMI < 18 kg/m^2^)	Healthy weight (BMI 18.5-24.9 kg/m^2^)	Overweight (BMI 25.0-29.9 kg/m^2^)	Obesity (BMI > 30 kg/m^2^)	*P*-value
	*N* = 82	*N* = 598	*N* = 326	*N* = 196	
**Demographics**					
Age (years)	23 ± 3^a,c,d^	24 ± 3^b,c,d^	25 ± 3^a,b,c^	25 ± 3^a,b,d^	**< 0.001**
Sex (Male, N, %)	45 (55)^a,b,c,d^	297 (50)^a,b,d^	163 (50)^a,c,d^	73 (37)^a,b,c,d^	**0.008**
Ethnicity (Black, N, %)	21 (26)^a,b,c,d^	299 (50)^a,b,c^	178 (55)^a,b,c,d^	98 (50)^a,c,d^	**< 0.001**
Socio economic status (score)	17.5 ± 5.48^a,b,c,d^	20.3 ± 5.98^a,b,c^	21.5 ± 6.03^a,b,c^	21.4 ± 6.41^a,d^	**< 0. 001**
**Lifestyle**					
Smoking (N, %)	30 (37)^a,b,c,d^	152 (26)^a,b,c^	58 (18)^a,b,c,d^	46 (24)^a,c,d^	**0.002**
Alcohol use (N, %)	50 (61)	315 (53)	189 (59)	112 (57)	0.244
**Body composition**					
Body height (cm)	168 ± 8.56	169 ± 8.98^a^	169 ± 10.3^b^	167 ± 16.9^a,b^	**0.022**
Body weight (kg)	49.5 ± 5.82^a,b,c,d^	62.4 ± 8.74^a,b,c,d^	77.9 ± 10.3^a,b,c,d^	96.1 ± 16.9^a,b,c,d^	**< 0.001**
Waist circumference (cm)	66.4 ± 5.45 ^a,b,c,d^	73.4 ± 6.33 ^a,b,c,d^	84.9 ± 7.19 ^a,b,c,d^	98.5 ± 11.9 ^a,b,c,d^	**< 0.001**
Body mass index (kg/m^2^)	17.6 ± 0.72 ^a,b,c,d^	21.8 ± 1.77 ^a,b,c,d^	27.2 ± 1.38 ^a,b,c,d^	34.6 ± 4.89 ^a,b,c,d^	**< 0.001**
**Cardiovascular measures**					
Office SBP (mmHg)	110 ± 11^a,b,c,d^	116 ± 12^a,b,c,d^	119 ± 11^a,b,c^	120 ± 11^a,b,d^	**< 0.001**
Office DBP (mmHg)	75 ± 7^a,c,d^	77 ± 8^b,c,d^	79 ± 7^a,b,c^	80 ± 7^a,b,d^	**< 0.001**
Pulse pressure amplification	1.48 ± 0.09^a,c,d^	1.47 ± 0.10^b,c,d^	1.45 ± 0.09^a,b,d^	1.39 ± 0.12^a,b,c,d^	**< 0.001**
Mean arterial pressure (mmHg)	86 ± 7^a,d^	86 ± 9^b,c,d^	88 ± 8^b,c,d^	91 ± 8^a,b,c,d^	**< 0.001**
Heart rate (bpm)	58 ± 10^a,c,d^	59 ± 9^b,c,d^	62 ± 9^a,b,c^	64 ± 9^a,b,d^	**< 0.001**
Pulse wave velocity (m/s)*	6.47 ± 0.86	6.36 ± 0.95	6.40 ± 0.98	6.19 ± 0.81	0.073
**Inflammation markers**					
Interleukin-6 (pg/ml)	1.70 (1.32;2.20)	2.24 (2.04;2.46)	1.98 (1.75;2.24)	1.92 (1.62;2.27)	0.101
Interleukin-8 (pg/ml)	1.83 (1.51;2.21)	1.92 (1.80;2.05)	1.77 (1.61;1.93)	1.64 (1.44;1.85)	0.107
Interleukin-10 (pg/ml)	4.26 (3.48;5.23)	5.13 (4.74;5.55)	5.08 (4.59;5.63)	4.28 (3.77;4.86)	0.060
Tumour necrosis factor-α (pg/mL)	1.01 (0.88;1.15)^a,d^	1.02 (0.97;1.06)^b^	1.08 (1.02;1.15)	1.22 (1.13;1.31)^a,b,d^	**< 0.001**
Adiponectin (μg/mL)	5.77 (4.99;6.68)^a,c,d^	4.64 (4.38;4.90)^b,c,d^	3.42 (3.14;3.70)^a,b,c,d^	2.63 (2.32;2.99)^a,b,c,d^	**< 0.001**
Leptin (ng/mL)	3.79 (2.90;4.75) ^a,b,c,d^	7.56 (6.86;8.59) ^a,b,c,d^	18.2 (16.4;21.9) ^a,b,c,d^	44.5 (39.9;52.1) ^a,b,c,d^	**< 0.001**
C-reactive protein (mg/L)	0.40 (0.29;0.55)^a,c,d^	0.53 (0.48;0.60)^b,c,d^	1.26 (1.09;1.44)^a,b,c,d^	3.29 (2.80;3.87)^a,b,c,d^	**< 0.001**
**Biochemical markers**					
Glucose (mmol/L)	4.83 ± 0.39^a,b,c,d^	4.98 ± 0.38^a,b,c,d^	5.11 ± 0.42^a,b,c,d^	5.24 ± 0.53^a,b,c,d^	**< 0.001**
Gamma-glutamyl transferase (U/L)	15.8 (13.8;17.8)^a,c,d^	15.8 (15.1;16.6)^b,c,d^	20.0 (18.2;21.4)^a,b,c,d^	25.7 (23.4;28.1)^a,b,c,d^	**< 0.001**
Total cholesterol (mmol/L)	3.46 ± 1.02^a,c,d^	3.61 ± 1.14^b,c,d^	3.89 ± 1.32^a,b,c^	4.12 ± 1.11^a,b,d^	**< 0.001**
HDL (mmol/L)	1.21 ± 0.42^a,d^	1.22 ± 0.43^b,c,d^	1.09 ± 0.43^b,c^	1.04 ± 0.35^a,b,d^	**< 0.001**
LDL (mmol/L)	2.21 ± 0.81^a,c,d^	2.26 ± 0.89^b,c,d^	2.59 ± 0.88^a,b,c,d^	2.82 ± 0.94^a,b,c,d^	**< 0.001**
Triglycerides (mmol/L)	0.69 ± 0.29^a,c,d^	0.74 ± 0.40^b,c,d^	0.91 ± 0.86^a,b,c,d^	1.11 ± 0.70^a,b,c,d^	**< 0.001**

Values are expressed as arithmetic means and standard deviation (for normally distributed data), geometric means with 5th and 95th percentiles (for non-normally distributed data), or proportions (for categorical data). Bold values denote statistically significant (*p* < 0.05) trends for analysis of variance. Values with the same superscript letters differ significantly from each other in group comparisons (*p* < 0.05). *Adjusted for mean arterial pressure.

In the supplementary analysis (Supplemental Table 1), the population was stratified according to WHtR categories. Similar findings to the BMI categories were evident, with PPA being lower in the increased WHtR group (*p* < 0.001), and markers of inflammation, including TNF-alpha, leptin and CRP being higher (all *p* < 0.001). No differences in PWV were evident between the groups (*p* = 0.776).

**Fig. 1:**
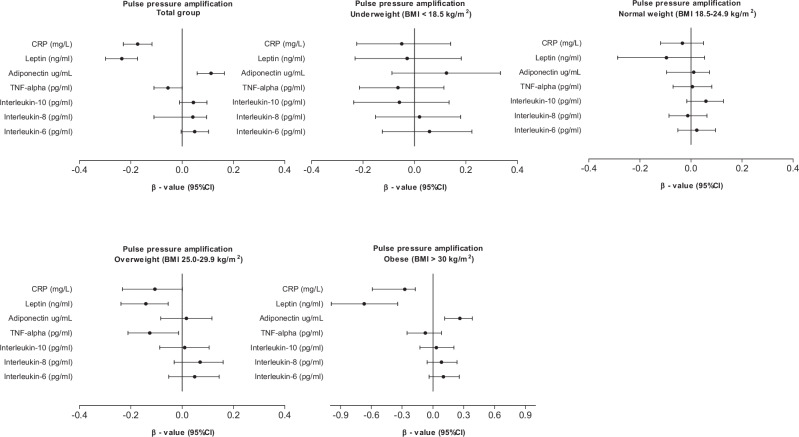
Associations between pulse pressure amplification and inflammatory markers in the total population and groups stratified according to body mass index. Models were adjusted for age, sex, ethnicity, socioeconomic status score, LDL, glucose, heart rate, smoking and alcohol use.

Figure 1 shows the associations between PPA and inflammatory markers in the total population and in groups stratified according to BMI, adjusted for age, sex, ethnicity, socioeconomic status score, LDL, glucose, heart rate, smoking and alcohol use. In the total group, PPA was negatively associated with CRP (β = -0.173, *p* < 0.001), leptin (β =-0.235, *p* < 0.001) and TNF-alpha (β = -0.055, *p* = 0.048), while a positive association with adiponectin (β = 0.113, *p* < 0.001) was found. Upon group stratification according to BMI, no associations between PPA and the inflammatory markers were evident in the underweight or healthy weight group. In the overweight group, PPA was negatively associated with leptin (β = -0.141, *p* = 0.002) and TNF-alpha (β = -0.125, *p* = 0,025), while a borderline association was seen for CRP (β = -0.106, *p* = 0.052). In the obesity group, PPA associated negatively with CRP (β = -0.274, *p* < 0.001) and leptin (β = -0.359, *p* < 0.001), while a positive association was found with adiponectin (β = 0.262, *p* < 0.001).

**Fig. 2:**
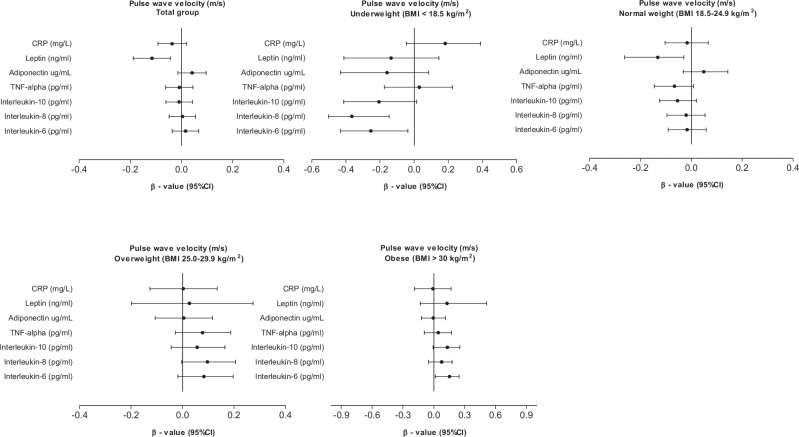
Associations between pulse wave velocity and inflammatory markers in the total population and groups stratified according to body mass index. Models were adjusted for age, sex, ethnicity, socioeconomic status score, LDL, glucose, heart rate, MAP, smoking and alcohol use.

Figure 2 shows the associations between PWV and inflammatory markers in the total population and in groups stratified according to BMI, adjusted for age, sex, ethnicity, socioeconomic status score, LDL, glucose, heart rate, smoking and alcohol use and MAP. Limited associations were evident, with PWV showing a negative association with IL-6 (β = -0.433, *p* = 0.021) and IL-8 (β = --0.365, *p* < 0.001) in the underweight group, while a weak negative association was also found with leptin the underweight group (β = -0.132, *p* = 0.015).

In supplementary analyses, the multiple regression models were repeated in groups stratified according to WHtR. In the healthy WHtR group (Supplemental Fig. 1), the only association evident was for PPA and leptin (β =-0.140, *p* = 0.004). In the high WHtR group, PPA was negatively associated with leptin (β =-0.293, *p* < 0.001) and CRP (β =-0.186, *p* < 0.001), while a positive association was seen with adiponectin (β = 0.130, *p* = 0.009). In Supplemental Fig. 2, the findings show that PWV was not associated with any of the inflammation markers in either WHtR group (all *p* > 0.117).

## Discussion

We compared PPA as well as an array of inflammatory markers in young adults stratified by body composition according to body mass index (BMI). We also determined the relationship of PPA and inflammatory markers within these groups. PPA was lower in the OW and OB groups, with the inflammatory profile also being worse in these groups. In fully adjusted regression models, no associations between PPA and the inflammatory markers were found in the underweight and healthy weight groups, while these markers were adversely associated with PPA in the OW and OB groups.

While it has been well-described that PP is a strong predictor of mortality in older populations [27–29], it is now also known that central PP may be a better indicator of CVD risk [30, 31] as this is the pressure associated with the pulsatile stress on the target organs such as the heart and brain [32]. Moreover, the amplification of PP from the central-to peripheral arteries due to a progressive increase in vascular stiffness, is also associated with cardiovascular risk [33] and data shows that an attenuation of PPA may be associated with unfavourable effects on both central arteries and the heart [34]. Our findings showed a lower PPA in the OW and OB groups, compared to the healthy weight and underweight groups, suggesting that an attenuation of PPA may already be taking place in these young adults. These findings are also supportive of previous data showing that higher levels of visceral fat correlate with greater aortic stiffness as measured by pulse wave velocity [35] and that aortic stiffness is positively associated with BMI and WC in elderly individuals [36]. More recently, an European study that also included apparently healthy men and women, showed PPA to be related to body fatness over a wide age-range [11].

Adiposity is associated with adverse changes in the arterial wall in both adolescents and adults [6, 7]. The pathophysiology behind a decreased arterial distensibility in an obese state, is multifactorial and involves hemodynamic, neurohumoral and metabolic factors [37]. Among the metabolic factors, hyperleptinemia may play an important role [37], with our findings also showing leptin to be progressively higher from the underweight to obesity group. In addition to leptin, adiponectin is an important role player in vascular homeostasis, by way of its atherogenic properties improving endothelial function and having anti-inflammatory effects in the vascular wall. In our current study, levels of adiponectin were progressively lower from the underweight to obesity categories, supporting the observations that its biosynthesis is deranged in obesity [38]. Further in the complex aetiology of obesity, neurohumoral activation may in turn also lead to systemic inflammatory processes [39] and obesity has extensively been described as an inflammatory condition [40]. Our findings support this classification by showing higher levels of inflammatory markers in the OW and OB groups, including increased TNF-alpha and CRP. In turn, chronic low-grade inflammation will have an effect on arterial structure and function by accelerating atherosclerosis, destabilizing plaques, impairing endothelial function, or causing premature arterial stiffness [41–43]. This possible disruption of vascular function is also evident in the adverse associations we showed between PPA and markers of inflammation (TNF-alpha, leptin, CRP and adiponectin) that were only evident in the OW and OB groups in models fully adjusted for traditional CV risk factors.

It is important to note that in many of the previous studies that investigated the interplay between arterial function and obesity, the measurement for the classification of obesity remains a topic of debate and an important consideration in terms of central vs peripheral obesity. Many researchers and clinicians make use of BMI as opposed to methods such as dual X-ray absorptiometry or computer tomography, due to its cost-effectiveness and simplicity of measurement and calculation [44], which can also be considered an important factor in resource-limited settings such as South Africa. However, central adiposity can still be estimated by measurement of waist circumference and the WHtR [45]. Taking this into account, we performed supplementary analysis exploring the differences in PPA and inflammatory markers as well as the independent relationships, in our study cohort stratified by WHtR. We encountered similar findings to those seen with the BMI classification, supporting the interplay between obesity and an attenuation in PPA, as well as adverse associations between PPA and inflammation in the setting of obesity.

The mechanism behind obesity and arterial function, as well as the possible mediators is important to understand as several outcomes of the obese state share a similar pathophysiological hallmark by way of vascular dysfunction leading to atherosclerotic plaque [46–48]. Arterial stiffness is often the result of cumulating damage on the arterial wall caused by CV risk factors (such as the detrimental effects of obesity and the associated inflammatory response) over an extensive period of time. While we did not encounter differences in PWV (as a direct measure of arterial stiffness) according to body composition, our findings on PPA may be indicative of systolic pressure amplification in young adults, beyond factors related only to arterial remodelling. It is conceivable that body composition and the associated inflammatory cascade, may alter factors such as peripheral vascular impedance, wave reflection and stroke volume, thereby affecting PPA, while these factors may not play a current role in large artery stiffness in this young population. Considering this, the identification of these factors and early intervention already in young adults, may provide an opportunity to prevent the development of clinically significant effects on target organs later in life.

The findings of this study should be interpreted within the context of some limitations. The cross-sectional design does not allow for the inference of causality. Participants were recruited from a specific municipal area of the North-West province and were between the ages of 20–30 years old, which is not representative of the entire young adult population. While adiposity was assessed using BMI and WHtR, which may not fully reflect the role of body fat percentage or visceral adiposity, these classifications are useful in resource-limited settings such as South Africa where direct measures are not always readily available. The strengths of our study lie in the inclusion of a relatively large sample size and the study was conducted under well controlled clinical conditions, providing insights into the understudied association between PPA, obesity and inflammation, especially in Africans.

In conclusion, in young South African adults, obesity is associated with a lower PPA and higher levels of inflammatory makers based on BMI and WHtR classification. A decrease in PPA, which is regarded as a surrogate for arterial stiffness, is associated with several inflammatory markers the setting of increased adiposity, highlighting the potential role of inflammation in the relationship between PPA and obesity.

## Summary

### What is known about the topic?

- PPA is an indirect measure of arterial elasticity and was shown to already be adversely affected in young populations.
- The degree of PPA is influenced by a number of physiological factors.

### What this study adds

- The contribution of low-grade systemic inflammation to PPA in young adults between 20-30 years is shown.
- The context of overweight and obesity in the relationship between PPA and inflammation in young adults is highlighted, with stratification by both BMI and WHtR.

## Supplementary information

Supplemental Table 1

Supplemental Figure 1

Supplemental Figure 2

## Data Availability

The dataset is available from the corresponding author on reasonable request.
