# Supplementary material for: Associations between pulse pressure amplification and inflammation in young adults according to body composition: The African-PREDICT study
Source: J Hum Hypertens. 2026 Feb 27;40(4):281–7. doi: 10.1038/s41371-026-01126-9 (PMC13068517; doi:10.1038/s41371-026-01126-9)
Supplement: Supplementary file 1 — Supplemental Table 1 [file 41371_2026_1126_MOESM1_ESM.docx]

| **Supplementary Table 1:** Basic characteristics of the study population stratified by waist-to-height ratio | | | |
| --- | --- | --- | --- |
|  | **Healthy**  **(WHtR < 0.5)** | **Increased**  **(WHtR ≥ 0.5)** | **P-value** |
|  | **N= 807** | **N = 394** |  |
| **Demographics** |  |  |  |
| Age (years) | 24 ± 3 | 25 ± 3 | **< 0.001** |
| Sex (Male, N, %) | 393 (49) | 184 (47) | 0.515 |
| Ethnicity (Black, N, %) | 391 (49) | 204 (52) | 0.279 |
| Socio economic status (score) | 20.3 ± 6.06 | 21.4 ± 6.16 | **0. 003** |
| **Lifestyle** |  |  |  |
| Smoking (N, %) | 194 (24) | 92 (23) | 0.775 |
| Alcohol use (N, %) | 433 (54) | 233 (59) | 0.079 |
| **Body composition** |  |  |  |
| Body height (cm) | 169 ± 9 | 167 ± 10 | **0.040** |
| Body weight (kg) | 64 ± 11 | 87 ± 17 | **< 0.001** |
| Waist circumference (cm) | 74 ± 7 | 93 ± 11 | **< 0.001** |
| Body mass index (kg/m^2^) | 22 ± 3 | 31 ± 5 | **< 0.001** |
| Waist-to-height ratio | 0.44 ± 0.03 | 0.56 ± 0.06 | **< 0.001** |
| **Cardiovascular measures** |  |  |  |
| Office SBP (mmHg) | 116 ± 12 | 120 ± 11 | **< 0.001** |
| Office DBP (mmHg) | 78 ± 8 | 80 ± 7 | **< 0.001** |
| Pulse pressure amplification | 1.47 ± 0.09 | 1.41 ± 0.11 | **< 0.001** |
| Mean arterial pressure (mmHg) | 86 ± 8 | 90 ± 8 | **< 0.001** |
| Heart rate (bpm) | 59 ± 9 | 63 ± 9 | **< 0.001** |
| Pulse wave velocity (m/s)* | 6.35 ± 0.92 | 6.37 ± 0.96 | 0.776 |
| **Inflammation markers** |  |  |  |
| Interleukin-6 (pg/ml) | 2.14 (0.28;11.7) | 1.69 (0.26;11.5) | 0.081 |
| Interleukin-8 (pg/ml) | 1.91 (0.48;7.94) | 1.66 (0.41;6.17) | **0.006** |
| Interleukin-10 (pg/ml) | 5.13 (1.26;21.9) | 4.57 (1.02;6.17) | **0.039** |
| Tumour necrosis factor-α (pg/mL) | 1.02 (0.41;2.39) | 1.15 (0.54;2.69) | **< 0.001** |
| Adiponectin (μg/mL) | 4.68 (1.28;12.8) | 2.81 (0.63;7.94) | **< 0.001** |
| Leptin (ng/mL) | 8.13 (6.60;45.7) | 28.1 (5.01;102) | **< 0.001** |
| C-reactive protein (mg/L) | 0.59 (0.07;5.89) | 2.08 (0.23;15.5) | **< 0.001** |
| **Biochemical markers** |  |  |  |
| Glucose (mmol/L) | 4.97 ± 0.39 | 5.21 ± 0.49 | **< 0.001** |
| Gamma-glutamyl transferase (U/L) | 15.8 (5.62;47.8) | 23.9 (8.32;83.2) | **< 0.001** |
| Total cholesterol (mmol/L) | 3.59 ± 1.12 | 4.09 ± 1.27 | **< 0.001** |
| HDL (mmol/L) | 1.21 ± 0.43 | 1.06 ± 0.37 | **< 0.001** |
| LDL (mmol/L) | 2.27 ± 0.89 | 2.78 ± 1.07 | **< 0.001** |
| Triglycerides (mmol/L) | 0.73 ± 0.46 | 1.06 ± 0.82 | **< 0.001** |
| Values are expressed as arithmetic means and standard deviation (for normally distributed data), geometric means with 5th and 95th percentiles (for non-normally distributed data), or proportions (for categorical data). Bold values denote statistically significant (p < 0.05) differences. *Adjusted for mean arterial pressure. | | | |
