# Supplementary material for: Associations between pulse pressure amplification and inflammation in young adults according to body composition: The African-PREDICT study
Source: J Hum Hypertens. 2026 Feb 27;40(4):281–7. doi: 10.1038/s41371-026-01126-9 (PMC13068517; doi:10.1038/s41371-026-01126-9)
Supplement: Supplementary file 3 — Supplemental Figure 2 [file 41371_2026_1126_MOESM3_ESM.pdf]

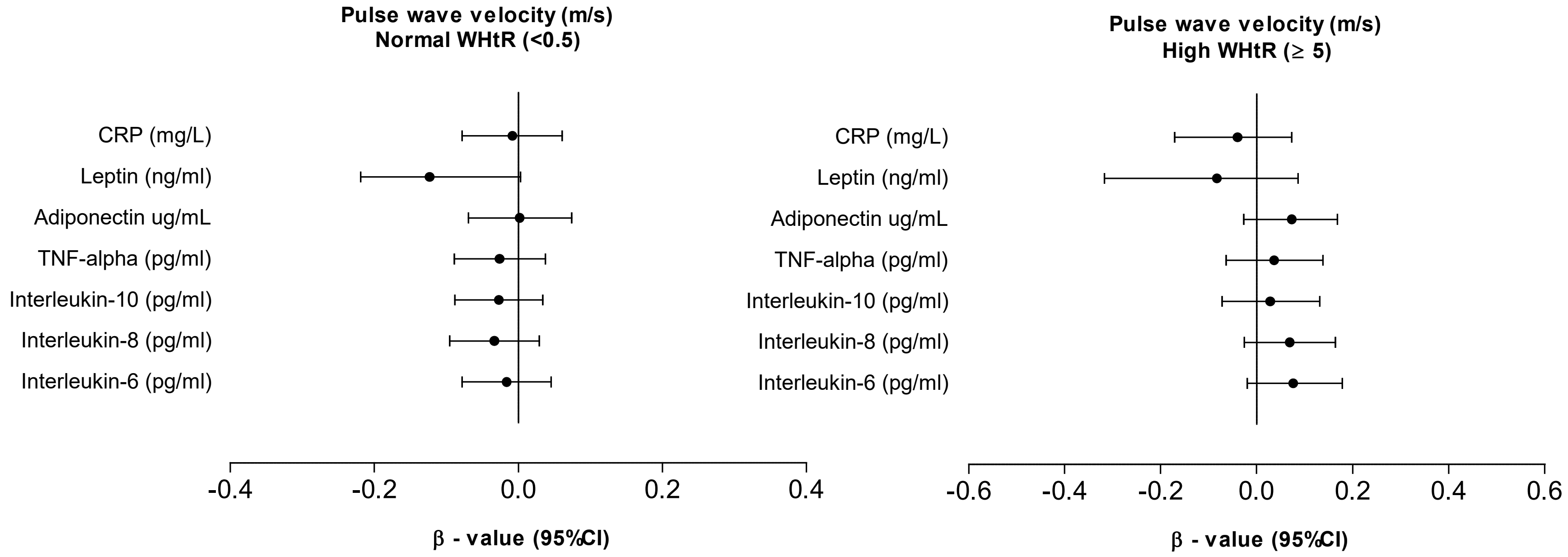

Supplemental Figure 2: Associations between pulse wave velocity and inflammatory markers in groups stratified according to waist-to-height ratio (WHtR). Models were adjusted for age, sex, ethnicity, socioeconomic status score, LDL, glucose, heart rate, MAP, smoking and alcohol use.
